# Body Size Plasticity of Weevil Larvae (*Curculio davidi*) (Coleoptera: Curculionidae) and Its Stoichiometric Relationship With Different Hosts

**DOI:** 10.1093/jisesa/ieaa139

**Published:** 2021-01-04

**Authors:** Baoming Du, Jun Yuan, Huawei Ji, Shan Yin, Hongzhang Kang, Chunjiang Liu

**Affiliations:** 1 School of Agriculture and Biology, Shanghai Jiao Tong University, Minhang, Shanghai, China; 2 Shanghai Urban Forest Ecosystem Research Station, State Forestry Administration, Minhang, Shanghai, China; 3 Key Laboratory of Urban Agriculture (South), Ministry of Agriculture, Minhang, Shanghai, China

**Keywords:** ecological stoichiometry, host–parasite interaction, homeostasis, body plasticity, fitness

## Abstract

Parasites obtain energy and nutrients from the host, and their body size is also usually limited by host size. However, the regulatory mechanisms that control the plasticity of parasite body sizes and the stoichiometric relationships with their hosts remain unclear. Here we investigated the concentrations of 14 elements (C, H, O, N, P, S, K, Na, Ca, Mg, Al, Fe, Mn, and Zn) in the acorns of three oak species (*Quercus* spp.), in their endoparasitic weevil (*Curculio davidi* Fairmaire) (Coleoptera: Curculionidae) larvae and in the larval feces, and the weight of weevil larvae within different hosts in a warm-temperate zone of China. Our results showed that the three acorn species exhibited significant differences in C, H, O, P, K, Mg, and Mn concentrations. However, in the weevil larvae, only P, Mn, and C:P ratio revealed significant differences. Weevil larvae preferentially absorbed and retained N, Zn, Na, and P, whereas Mn, K, Ca, and O were passively absorbed and transported. The weevil larvae weight was associated with acorn stoichiometry, and positively correlated with acorn size. Weevil larvae P decreased, but Mn and C:P increased with their weight, implying highly variable in somatic stoichiometry are coupled with the plasticity of body size. Interestingly, weevil larvae weight was negatively correlated with acorn infection rate, indicating small-size parasitic insects might have higher fitness level in parasite–host systems than larger-size ones. Our results suggest that variation in P, Mn, and C:P in parasites may play critical roles in shaping their body size and in improving their fitness.

Significant elemental imbalances exist between food consumers and available resources, including plants and insects (Sterner and Elser 2002, Cherif and Loreau 2013, Filipiak and Weiner 2017a). These imbalances, which are primarily caused by the level of food quality, may affect insect growth, population dynamics, and competition with other species (Muller et al. 2001, Moe et al. 2005, Filipiak and Weiner 2017b). As a theory for the study of the mass balance of multiple elements in ecological processes, ecological stoichiometry provides an integrative approach for understanding energy and elemental fluxes across different trophic levels (Sterner and Elser 2002). Homeostatic regulation, however, as a central concept of ecological stoichiometry, indicates that organisms attempt to sustain internal element concentrations as much as possible when the environments change (Karimi and Folt 2006, Persson et al. 2010, Jeyasingh et al. 2017). Previous studies have demonstrated that herbivorous insects can maintain stoichiometric homeostasis through a set of mechanisms, such as pre-ingestion (selective feeding or greater food consumption), post-ingestion (differential assimilation or excretion), or a combination of both (Cease et al. 2016, Steward et al. 2019). Usually, macronutrients are strongly regulated by organisms, whereas essential micronutrients are weakly regulated (Karimi and Folt 2006). Since endoparasitic insects live within their food sources, they are exclusively dependent upon their hosts. It has been reported that the interactions between parasites and hosts might influence host fitness, the stability of food-webs, and even nutrient cycling in ecosystems (Lafferty et al. 2008, Brunner and Eizaguirre 2016, Vannatta and Minchella 2018). However, little is known regarding the nutritional relationships between parasites and hosts, or the homeostasis mechanisms of parasites.

Since body size is plastic, the final body size of an insect results from the interactions between genetic and environmental factors (Blanckenhorn 2000, Mirth et al. 2016). It has been demonstrated that insects reared on poor-quality diets are smaller, whereas those reared at lower temperatures were larger than controls (Davidowitz et al. 2003). In terrestrial systems, N and P-mediated limitations on growth may be very common among insects (Elser et al. 2000) (e.g., N-limitation in *Tuta absoluta* [Coqueret et al. 2017] and P-limitation in *Schistocerca Americana* [Cease et al. 2016]). Small insects with a high body P content are more likely to be P-limited in food resources than large ones, which relates to a higher growth rate in small insects that have a higher requirement for P-rich ribosomal RNA (Woods et al. 2004, Hambäck et al. 2009). Previous studies also revealed that body P content was negatively correlated with body size (Hambäck et al. 2009, Paseka and Grunberg 2019). Moreover, somatic growth dilution can trigger elemental changes in organisms (Karimi et al. 2010). In parasitic systems, however, insect body sizes are constrained by both the available food resources and morphological dimensions of their hosts. Therefore, the evolutionary mechanism of body size in parasites remains unclear, particularly in regard to the relationship between body size plasticity and the nutrients related to insect growth, such as N and P as well as some mineral nutrients.

Acorn weevil larvae are a common predispersal parasite of oak (*Quercus* spp.) trees in the Northern Hemisphere (Aldrich and Cavender-Bares 2011) that complete their development within a single acorn (Bonal and Muñoz 2008). This is also a key life-history trait for most parasitic insects. Within the differently sized acorns of oak species, the sizes of weevil species were also observed to differ (Munoz et al. 2014). Interestingly, even within the same weevil species, individual larval weights were also variable between acorn species, independent of acorn size (Muñoz et al. 2014). Ordinarily, smaller acorn sizes reduce the availability of food; thus, constraining larval size, as the acorns do not provide sufficient food for the weevils to attain their full growth potential (Bonal and Muñoz 2009). Further, not only acorn size but also their chemical compositions influence weevil growth and population dynamics (Siscart et al. 1999, Sun et al. 2015). Being larger and fitter, but always within the constraints of available host sizes, may be one of the primary evolutionary dilemmas for endoparasites (Bonal and Muñoz 2008).

For this study, we investigated three natural oak stands *Quercus aliena* var. *acutiserrata*, *Quercus glandulifera*, and *Quercus variabilis* at three sites in the Baotianman Natural Reserve Henan Province, Central China. Three acorns and their typical parasitic weevil larvae (*Curculio davidi*) samples were collected at each site. The chemical compositions of the acorns, weevil larval, and larvae feces were measured and the relationships between the homeostasis and body sizes of the parasites were evaluated. Our objective was to explore how the weevil larvae that resided within different acorn species (i.e., their nutrient sources) and acorn sizes maintained their homeostasis and adjusted their body sizes. Specially, we asked: 1) How do parasites regulate and maintain homeostasis in the face of variable nutrient supplies? 2) How do hosts limit the growth of parasites through body size and nutrient availability, and which factors may play key roles in shaping the body sizes of parasites?

## Methods

### Study Area

This study was carried out at the Forest Ecological Research Station, in the Baotianman Natural Reserve (111°47′–112°04′ E, 33°20′–33°36′ N), of Henan Province, in Central China, which is located in a temperate-subtropical ecotone. The area has a warm-temperate climate with an average elevation of 1,350 m. The annual mean precipitation and air temperature are 900 mm and 15.1°C, respectively (Liu et al. 2017). The main soil type is haplic luvisol, and the forests consist primarily of deciduous oaks, including *Q. aliena* var. *acutiserrata*, *Q. glandulifera*, and *Q. variabilis*, along with minor components of some deciduous woodland species and shrubs.

### Sampling

Three sites were selected in *Q. aliena* var. *acutiserrata*, *Q. glandulifera*, and *Q. variabilis* forests, respectively, with each site representing a stand of the subject tree. At each site, three 20 × 20-m plots were established, where a total of 15 trees (5 trees for each plot) were selected. Six seed traps were placed symmetrically in the understory of each plot for acorn collection. Each trap contained a PVC frame (1-m L ×1-m W×1.2-m H) that was covered and secured with 2-mm nylon mesh. The bottoms of the traps were 0.5–0.8 m above the surface of the ground to discourage the removal of the seeds by animals. The acorns of the three oak species were collected every 5 or 7 d, from September to November, and then transferred to the laboratory. The establishment of the plots was partly based on the ‘Observation Methodology for Long-term Forest Ecosystem Research’, from the National Standards of the People’s Republic of China (GB/T 33027-2016).

Three plots were established for each oak species and ~1 kg of acorns was collected from each plot. The acorns from each plot were divided into three parts. First, 30 mid-sized (Supp Fig. 1a [online only]) and healthy acorns were selected from each plot to make a composite sample for chemical analysis (*n* = 3 for each acorn species). Second, 200 mid-sized and wormhole-free acorns from each plot were weighed and then transferred to a PVC tray to incubate the weevil larvae (*C. davidi*; Sun et al. 2015; Supp Fig. 1b [online only]). Under natural conditions, the larvae are limited to a single acorn and require about one month to complete their development, after which the fifth-instar larvae (Sun et al. 2015) emerge and burrow into the soil until the pupation in July of the second year. Herein, weevil larvae were incubated for 30 d in the laboratory at 25°C. The fifth-instar larvae were collected every 12 h and then individually weighed. The 200 acorns were opened with a pliers after 30 d to calculate the infection rate (the number of infected acorns divided into the total number of acorns). Third, the remaining (~1 kg) acorns were placed in a PVC tray to collect the weevil larvae (Supp Fig. 1c [online only]). The fifth-instar larvae and feces were collected every 12 h for 30 d, and then frozen and stored for chemical analysis. Following their collection, all larvae and all fecal samples were combined from a given plot to create a composite sample. The acorn and fecal samples were dried at 65°C for 72 h. Once the shells of the acorns were removed, the samples were ground with a Tecator sample mill (Subang, Shanghai, China), and the fecal samples with a ceramic mill, prior to being sieved through a 60-mesh sieve (0.25-mm diameter) for chemical analysis. The larval samples were ground with liquid nitrogen due to their high grease content, and then dried at 60°C for 96 h for chemical analysis.

### Chemical Analysis

The total C, H, O, and N concentrations in the acorns, weevil larvae, and feces were determined via elemental analysis (Vario EL cube, Elementar, Germany). Once all of the samples (acorn, weevil larvae, and feces) were digested with nitric and chloric acid, the total P, S, K, Na, Ca, Mg, Al, Fe, Mn, and Zn were analyzed with an inductively coupled plasma optical emission spectrometer (ICP-OES; iCAP6300, Thermo). Elemental analyses were performed at the Instrumental Analysis Center of Shanghai Jiao Tong University.

### Estimation of Nutrient Demand in the Parasites

The nutrient uptake ratio (*NUR*) is defined as below after Whitehead (2000),

$$\begin{array}{l} \mathrm{NUR}=\frac{EC_{food}}{EC_{insect}}\;\;\; \end{array}$$ (1)

where *E*_food_ and *E*_insect_ are the elemental concentration (EC) in acorns and weevil larvae, respectively. If *NUR* was > 1, the concentration of elements from the insect was greater than that of the acorns, which suggested that the element was preferentially absorbed and retained by the parasite. If *NUR* was <1, this indicated that the concentration of elements in the parasite was lower than that of the acorns, which signified that the parasite may have selectively limited the absorption of the certain element. In other words, the available element exceeded the requirements of the parasite.

The nutrient release ratio (*NRR*) is defined as below

$$\begin{array}{l} NRR=\frac{EC_{feces}}{EC_{insect}}\;\;\; \end{array}$$ (2)

where *E*_feces_ and *E*_insect_ are the elemental concentration (EC) in the weevil larvae and feces, respectively. When *NRR* was >1, the concentration of a given element in the feces was higher than that in the weevil, which suggested that the parasite was extracting this element, while disregarding other nutrients. When *NRR* was <1, this indicated that the concentration of a given element in feces was lower than that of the weevil, which suggested that it was being preferentially absorbed and retained by the parasite.

### Data Analysis

The concentrations of the elements were log_10_ transformed to improve normality. One-way analysis of variance (ANOVA; Duncan’s multiple range test selected) was employed to examine the differences of element concentrations between the acorns, larvae, and feces. Linear regressions were performed to correlate between the weevil larvae weights and acorn size, as well as acorn nutrient and weevil nutrient. To assess the influences of host sizes and nutrients on weevil size and weevil weight, as well as host sizes and nutrients on weevil P, we performed a stepwise multiple regression analysis. All analyses were carried out using SAS 8.1 (Institute Inc., Cary, NC) and SPSS 16.0 (IBM, Chicago, IL) software. Figures were drawn using SigmaPlot 10.0 (Systat Software, Inc., Richmod, CA).

## Results

### Stoichiometric Compositions of Acorns and Weevil Larvae

**Fig. 1.**
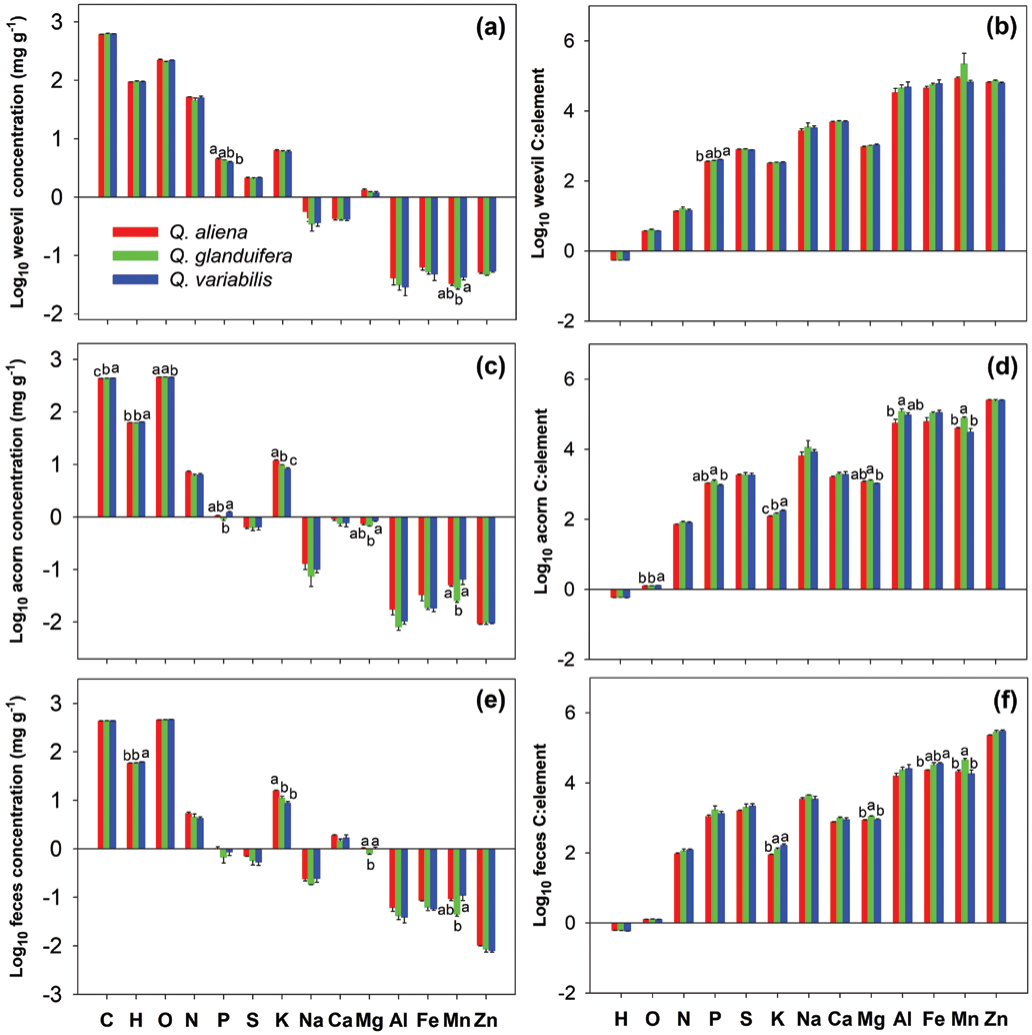
Element concentrations (a, c, and e) and C:element molar ratios (b, d, and f) of acorns, weevil larvae, and feces in three oak species (*Quercus aliena* var. *acuteserrata; Quercus glandulifera*, and *Quercus variabilis,* respectively). Different letters above bars represent a significant difference at the *P* < 0.05 level, by Duncan’s multiple range test (*n* = 9).

**Fig. 2.**
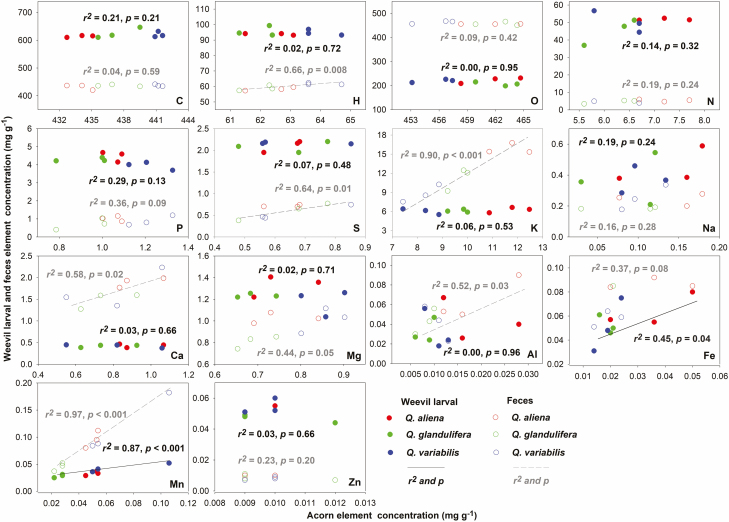
Relationships between weevil larvae and feces element concentrations and acorn element concentrations for three oak species.

The three acorn species exhibited significant elemental and stoichiometric differences in C, H, O, P, K, Mg, and Mn concentrations, and C:O, C:P, C:K, C:Mg, C:Al, and C:Mn ratios (*P* < 0.05; Fig. 1). In the weevil larvae, only P, Mn, and C:P ratios revealed significant differences (*P* < 0.05; Fig. 1). In the parasite feces, H, K, Mg, and Mn concentrations, and C:K, C:Mg, C:Fe, and C:Mn ratios were significantly affected by changes in the acorns (*P* < 0.05; Fig. 1). The Fe and Mn concentrations in the weevil larvae increased with the acorn nutrients (*P* < 0.05), as did H, S, K, Ca, Al, and Mn in the feces also increased with acorn nutrients (Fig. 2).

### Evaluation of Nutrient Demand and Metabolism

**Fig. 3.**
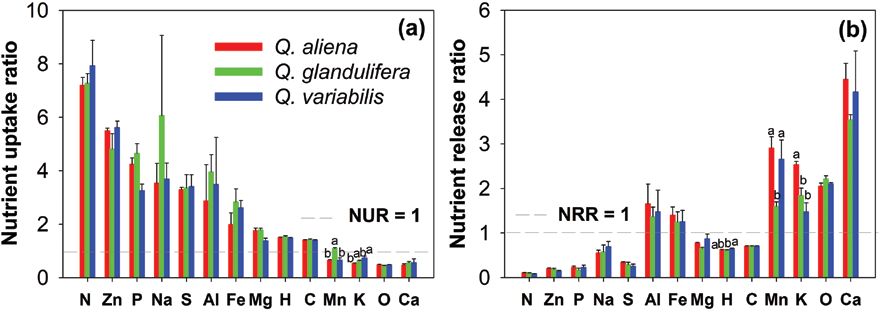
NUR (a) and NRR (b) of weevils feeding on three different oak acorn species. The NUR is equal to the concentrations of elements in the weevil larvae divided by the concentrations of elements in the acorns. The NRR is equal to the concentrations of the elements in the weevil larvae feces of the weevils divided by the concentrations of the elements in the weevil larvae itself. Lowercase letters indicate a significant difference at *P* < 0.05 by Duncan’s multiple range test.

The concentrations of O, K, Ca, and Mn in the weevil larva were significantly lower in contrast to their nutrient hosts, whereas the other 10 elements (with the exception of Mn, Ca, and Al in *Q. variabilis*) were greater (Supp Table 1 [online only]; *P* < 0.05). Nitrogen NUR (>7) was the greatest among the 14 elements; Zn, P, Na, and S NURs were also > 3; C, H, and Mg NURs were close to 1; whereas the weevil larvae had low Ca, O, K, and Mn requirements, the NURs were <1 (Fig. 3a). Compared with nutrient uptake, Ca, Mn, K, O, Al, and Fe were easily excreted in the feces, which showed that their NRRs were >1, while other elements were preferentially retained by the weevil larvae, revealing that the NRRs were <1 (Fig. 3b).

### Effects of Acorn Size and Nutrition on Weevil Larvae Body Size, and Their Relationship With Somatic Nutrients

**Fig. 4.**
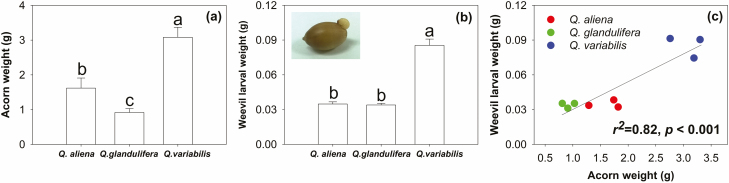
The weights of acorns and weevil larvae, and the relationships between the weevil larvae and acorn weights.

**Fig. 5.**
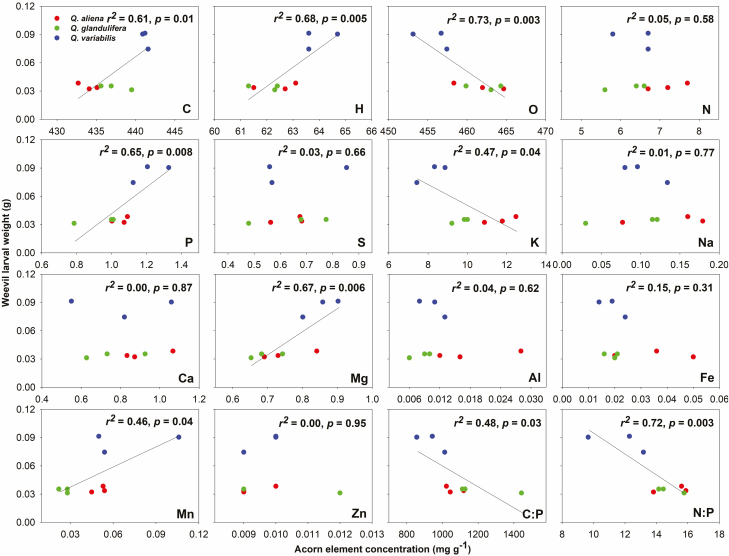
The relationships between acorn stoichiometry and weevil weight.

**Fig. 6.**
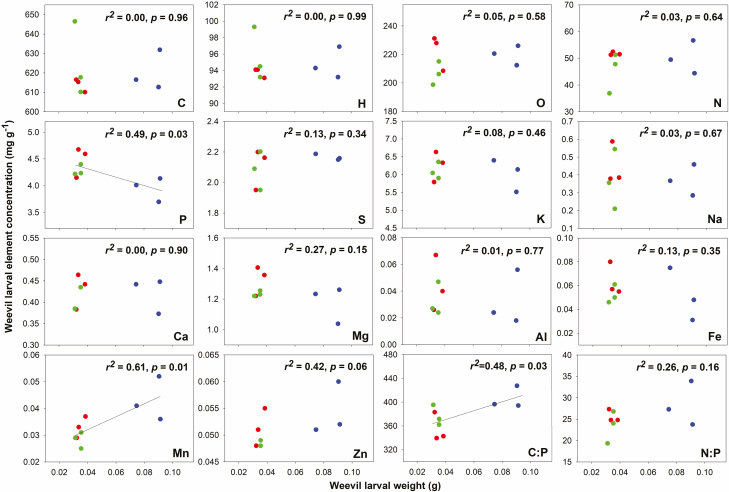
The relationships between weevil stoichiometry and weevil weight.

The weight of *Q. variabilis* acorns was highest among the three acorn species, and for the remaining two acorn species, the weight of *Q. aliena* was significantly higher than that of *Q. glandulifera* (Fig. 4a). The weevil larval weight in *Q. variabilis* acorns were significantly different than those of *Q. aliena* and *Q. glandulifera*, which showed no differences (Fig. 4b). The weights of the weevil larvae in the oak species increased with acorn weights (Fig. 4c). Meanwhile, the weevil larval weight was positively correlated with acorn C, H, P, Mg, and Mn, but negatively with O, K, and the C:P, and N:P ratios (Fig. 5). Moreover, the concentrations of P in weevil larvae were reduced, whereas Mn and the C:P ratio increased with their weight, while no relation was observed with the other elements (Fig. 6).

### Host Infection Rate and Its Relationship With Weevil Larvae Body Size

**Fig. 7.**
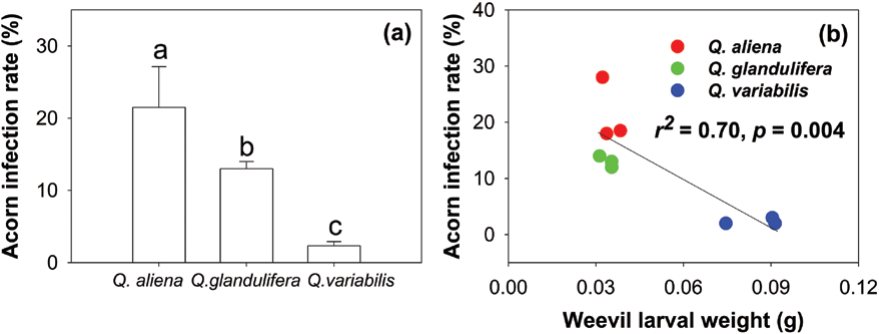
The infection rate of three acorn species, and the relationship between weevil weight and infection rate.

The infection rate of *Q. aliena* acorns was highest between the three acorn species, where in the remaining two acorn species, the infection rate of *Q. glandulifera* was significantly higher than that of *Q. variabilis* (Fig. 7a). By stepwise regression analysis, acorn infection rate was significantly related to the weevil larval weight (Supp Table 2 [online only]), and there was negative correlation between them (Fig. 7b).

## Discussion

Since weevil larva are confined to a single acorn, they cannot balance their diet by consuming other nutrients. When weevil larvae encounter acorns with different qualities, they form strong physiological postingestion patterns to maintain the regulation of chemical balances through nutrient uptake and release. However, due to the constraints imposed by host spaces, parasitic insects must adjust their body size plasticity to adapt to these habitats, which likely influences parasite fitness.

### Imbalanced Parasite/Host Nutrient Relationships

The quantification of limiting elements, and excess of nonlimiting elements, as well as the correct prediction of their fates remain problematic (Anderson et al. 2005). In this study, NUR and NRR were employed to explore the nutrient relationships between the acorn hosts and their weevil larvae parasites. Weevil larvae had the greatest N uptake ratio among the 14 elements, which indicated that acorn N could be the most limited element for the larvae. This was verified by the Trophic Stoichiometric Ratio Index (TSR) method described by Filipiak (2018; for detailed methods and results, see Supp Fig. 2 [online only]). For other elements, such as Zn, Na, P, Al, and S, the weevil larvae also showed high nutrient requirements (NUR > 1; Fig. 3). The results suggested the elements as well as N were preferentially concentrated in the weevil larvae. Typically, elemental N, P, and S are important components of nucleic acids/proteins in organisms, which are essential for insect growth and reproduction. Mineral nutrients, which play important roles in enzymes and ionic balance are also essential for insects (Soetan et al. 2010, Simpson and Chapman 2013). Correspondingly, however, weevil larvae had low O, Ca, K, and Mn requirements, where NURs were <1. This meant that the nutrients were well supplied, in other words, they were easily excreted via the feces (Fig. 3). Since weevil larvae as invertebrates do not require the high Ca levels required for bone growth, they have low Ca concentrations. Low O concentrations in parasites are likely related to energy reserves. This is because weevil larvae store energy in low O lipids, rather than high O carbohydrates (Sun et al. 2013). Lipids have the capacity to provide more energy than carbohydrates, which can enhance parasite the resistance of parasites against cold, desiccation, and damp conditions during the winter months (Burt et al. 2012). On the one hand, our results clearly reflected the differences in nutrient composition between plants and insects, on the other hand, also suggested that the demand for nutrients in weevil larvae is selective, which is likely driven by the growth requirements of insects.

However, in different forest ecosystems, weevil larvae have different nutrient demand. As estimated using data from Ji et al. (2017), we found that weevil larvae parasitized from *Q. variabilis* and *Q. acutisssima* acorns had similar NUR (Supp Fig. 1 [online only]; Ji et al. 2017), which aligned well with our results. This verified an interesting finding that local weevil larvae show similar NUR regardless of the acorns that they consume. However, Ji et al. (2017) revealed in their study that weevil larvae had a high nutrient P uptake, rather than the N that we found. These results suggested that weevil may be more likely to be restricted in N by host acorns (as in our study), while in subtropical areas they may be more limited in P. This could be caused by host nutrient because limiting nutrients are usually retained at greater efficiencies by the consumer (Cross et al. 2003). Essentially, in our study, acorns did indeed have lower N and higher P in contrast to subtropical acorns (Supp Table 3 [online only]). The results imply that parasitic insects feeding on different hosts may show similar nutritional demands at the localized scales; however, at large spatial scales, these demands would be modified in accordance with changes in host nutrients and environmental conditions (Sun et al. 2012, 2013, 2015).

For the present study, we ask how weevil larvae that develop in three different acorn species with varying nutritional resources might systematically regulate their stoichiometric balances? This, in view of the fact that the regulation of homeostasis for organisms is a complex process. Based on nutrient uptake and nutrient release ratios, we found that there might be a reciprocal pattern for the regulation of chemical balance in insects. On one hand, highly required nutrients were the greatest of the preferentially retained elements as, in terms of nutrients, insects typically possessed high NUR and low NRR in terms of nutrients (Fig. 3). Conversely, for excess (i.e., low-requirement) nutrients such as Ca, K, Mn, and O, they were more easily discriminated through fecal excretion, which resulted in insects generally having lower NUR and higher NRR for these. Similar findings have also been reported in other studies (Anderson et al. 2005, Frost et al. 2005). Our findings indicated that to achieve optimal nutrient requirements, insects might engage a trade-off mechanism to regulate the balance of multiple elements. Thus, increased absorption and reduced excretion for high-requirement nutrients would occur through this mechanism, while for nonlimiting elements, reduced absorption and enhanced release would ensue.

### Regulation of Homeostasis and Plasticity of Body Size

Our results revealed that although the chemical compositions of the three acorn species varied significantly, the stoichiometry of the weevil larvae was not affected, with the exception of P, Mn, and C:P (Fig. 1). This aligned with the study of Ji et al. (2017), who found that there were stoichiometric differences between the two acorn species, but not between weevil larvae. These results suggested that parasitic insects may have more stable stoichiometric compositions compared to their hosts, as demonstrated in previous studies (Moe et al. 2005, Liu and Sun 2013).

It is well known that manganese (Mn) is a toxic element, particularly in terms of neurotoxicity (Ben-Shahar 2018). In this study, the low-stability of Mn was related to nutrient uptake and release processes. We found that Mn concentrations in weevil larvae increased with acorn Mn, whereas those in larval feces were even higher (Fig. 2). These results suggested that Mn might simply be passively stored and transported; thus, it be a ‘disregarded’ element during the nutrient regulation process. Another possibility is that weevil larvae systems attempt to maintain Mn homeostasis (fecal Mn concentrations increases with higher acorn Mn concentrations); however, the variations in Mn may be too great for them to overcome through this mechanism. Martinek et al. (2017) also reported similar findings when leaf weevils (*Phyllobius arborator*) were fed on food high in Mn, they avoided Mn damage by increasing excretion. Interestingly, this stable Mn relationship between the consumer and nutrient source was completely different from previous findings, which may have important implications for the study of nutrient flow in plant–insect interactions.

It has been observed that phosphorus (as a nonstrict homeostasis nutrient) might increase insect fitness through the regulation of homeostasis (Schade et al. 2003). In the present study, we observed that large acorn size and high acorn quality can indeed stimulate insect growth; however, due to the limitations of acorn space, it is obviously impossible for insects to grow unrestrained. Therefore, when insects reside within large acorn species, they must reduce the P investments in their body systems to constrain growth (Fig. 6). In other words, the smaller the insect, the higher the P content in its body. It has also been demonstrated in previous studies that smaller species possess higher metabolism and P content than larger species (Paseka and Grunberg 2019), presumably because the higher growth rate in smaller species places a higher demand on P-rich ribosomal RNA (Hambäck et al. 2009). Another explanation is likely due to the dilution effect of somatic growth (Karimi et al. 2010). Larger acorns contain larger weevil larvae, which might lead to the dilution of body P content with increasing insect body size. Our findings indicated that the regulation of P homeostasis played a critical role in larval adaptations to different acorn nutrient conditions and host sizes. Further, because P was directly related to insect growth, variations of P in parasitic insects were also coupled with the plasticity of acorn body sizes of the three oak species.

However, based on the infection rate data of the three oak acorn species (Fig. 7a), we found that, in terms of the plasticity of body size, the larger bodies of parasitic insects did not correlate with their fitness. The acorns of *Q. variabilis* are large and rich in nutrients, which benefited the growth of parasite insects; however, large acorn bodies did not improve weevil larval fitness, as their infection rate was actually the lowest. In contrast, small-bodied parasites resulting from small acorn sizes had greater benefits for survival and development (Fig. 7). This was likely due to the constant P content in the weevil larvae, where small-bodied insects played an important role in reducing the consumption of C (Fig. 6), which assists insects with storing energy and improving resistance against low temperatures. This responded further to: ‘The evolution of body size: what keeps organisms small?’, raised by Blanckenhorn (2000). However, our study supported this question solely from the aspects of food selection, nutrients, and habitat space. The roles of other factors, such as plant defences and plant–parasite–animal interactions warrant further research.

In conclusion, seed-parasitic insects always encounter the hosts with variable nutrients and different size accommodation habitats from distinct plant species. Our results confirmed that the postingestion mechanisms not only regulated the somatic elemental balance in parasitic insects but can also regulate their body size within the host. The regulation of dual postingestion, stoichiometry and life-history traits appear to be linked: 1) High nutrient requirements show a high uptake and low release ratio pattern, while low nutrients requirements are linked with a low uptake and high release ratio pattern (Trade-off Hypothesis of Nutrient Regulation). 2) Compared with their hosts, parasitic insects exhibit a much more robust homeostatic control of elements, except for somatic P. The underlying mechanisms include that highly variable in somatic P, Mn, and C:P ratios are coupled with the plasticity of body size, which benefits parasitic insects to adapt to hosts with variable nutrients and body sizes. 3) Diminutive parasitic insects might have higher fitness levels in parasite–host systems than their larger counterparts. Our results contribute novel insights toward further elucidating coexisting parasite–host systems in terms of ecological stoichiometry.

## Supplementary Material

ieaa139_suppl_Supplementary_Materials
