# Supplementary material for: Body Size Plasticity of Weevil Larvae (Curculio davidi) (Coleoptera: Curculionidae) and Its Stoichiometric Relationship With Different Hosts
Source: J Insect Sci. 2021 Jan 4;21(1):2. doi: 10.1093/jisesa/ieaa139 (PMC7780276; doi:10.1093/jisesa/ieaa139)
Supplement: ieaa139_suppl_Supplementary_Materials [file ieaa139_suppl_supplementary_materials.docx]

**Supplementary Data**

**Body size plasticity of weevil larvae (*Curculio davidi*) and its stoichiometric relationship with different hosts**

Baoming Du^1,2^, Jun Yuan^1^, Huawei Ji^1,2^, Shan Yin^1,2,3^, Hongzhang Kang^1,2,3^, Chunjiang Liu^1,2,3,4^

^1^School of Agriculture and Biology, Shanghai Jiao Tong University, Minhang, Shanghai 200240, China, ^2^Shanghai Urban Forest Ecosystem Research Station, State Forestry Administration, Minhang, Shanghai 200240, China, ^3^Key Laboratory of Urban Agriculture (South), Ministry of Agriculture, Minhang, Shanghai 200240, China, ^4^ Correspondence author, e-mail: [chjliu@sjtu.edu.cn](mailto:chjliu@sjtu.edu.cn)


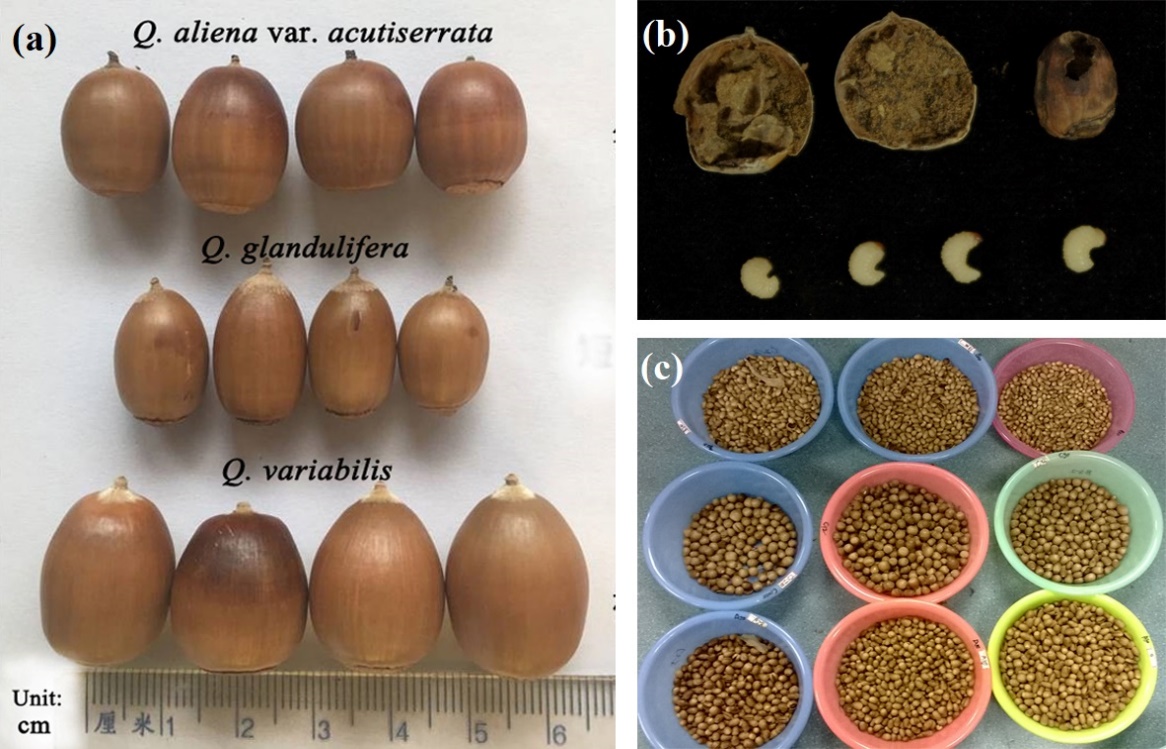


**Fig. S1.** Acorns (a) and weevil larvae (b) morphology and sampling scene (c).

**Table S1** Element concentrations of acorns, weevil larva, and feces for three oak species.

| Elements | *Q. aliena* (var.) | | |  | *Q. glandulifera* | | |  | *Q. variabilis* | | |
| --- | --- | --- | --- | --- | --- | --- | --- | --- | --- | --- | --- |
|  | Acorns | Weevil larvae | Feces |  | Acorns | Weevil larvae | Feces |  | Acorns | Weevil larvae | Feces |
| C (mg g^-1^) | 433.97±0.70b | 613.97±1.96a | 431.23±5.47b |  | 437.33±1.15b | 624.80±11.1a | 436.67±2.10b |  | 441.23±0.20b | 620.37±5.87a | 437.07±2.17b |
| H (mg g^-1^) | 62.43±0.48b | 93.77±0.33a | 58.33±0.64c |  | 62.00±0.35b | 95.67±1.86a | 58.90±0.96b |  | 63.97±0.37b | 94.80±1.10a | 61.63±0.33b |
| O (mg g^-1^) | 461.66±1.84a | 222.57±7.10b | 455.73±0.26a |  | 462.41±1.32a | 206.66±4.71b | 457.10±4.5a |  | 455.76±1.33a | 219.70±3.97b | 463.26±3.54a |
| N (mg g^-1^) | 7.20±0.29b | 51.73±0.34a | 5.37±0.35c |  | 6.20±0.31b | 45.33±4.34a | 4.60±0.60b |  | 6.40±0.30b | 50.20±3.57a | 4.23±0.33b |
| P (mg g^-1^) | 1.06±0.03b | 4.47±0.16a | 1.02±0.09b |  | 0.93±0.07b | 4.28±0.06a | 0.72±0.18b |  | 1.22±0.06b | 3.95±0.13a | 0.89±0.16b |
| S (mg g^-1^) | 0.64±0.04b | 2.10±0.08a | 0.72±0.01b |  | 0.64±0.09b | 2.08±0.07a | 0.60±0.12b |  | 0.66±0.10b | 2.17±0.01a | 0.55±0.10b |
| K (mg g^-1^) | 11.71±0.46b | 6.25±0.25c | 15.83±0.47a |  | 9.69±0.24a | 6.10±0.13b | 11.25±1.03a |  | 8.20±0.42a | 6.02±0.26b | 8.76±0.79a |
| Na (mg g^-1^) | 0.14±0.03b | 0.45±0.07a | 0.24±0.02b |  | 0.09±0.03b | 0.37±0.10a | 0.19±0.00ab |  | 0.10±0.02b | 0.37±0.05a | 0.25±0.05b |
| Ca (mg g^-1^) | 0.92±0.07b | 0.43±0.02c | 1.90±0.07a |  | 0.76±0.09b | 0.42±0.02c | 1.49±0.11a |  | 0.81±0.15b | 0.42±0.02b | 1.71±0.27a |
| Mg (mg g^-1^) | 0.75±0.04c | 1.33±0.06a | 1.03±0.03b |  | 0.69±0.03c | 1.24±0.01a | 0.81±0.03b |  | 0.85±0.03b | 1.18±0.07a | 1.01±0.07ab |
| Al (mg kg^-1^) | 18.70±4.81b | 44.30±12.0ba | 64.30±12.9a |  | 8.33±1.20b | 32.70±7.22a | 43.00±7.51a |  | 10.70±1.45a | 32.70±11.80a | 41.70±10.20a |
| Fe (mg kg^-1^) | 35.30±8.67b | 64.00±8.02a | 87.00±2.52a |  | 19.00±1.53b | 52.30±4.48a | 64.70±10.80a |  | 19.00±2.89b | 51.30±12.8a | 58.00±3.79a |
| Mn(mg kg^-1^) | 50.70±2.85b | 33.00±2.31b | 95.70±9.24a |  | 26.00±2.00b | 28.3±1.76b | 45.30±4.41a |  | 70.00±18.00a | 43.00±4.73a | 118.00±32.00a |
| Zn (mg kg^-1^) | 9.33±0.30b | 51.30±2.03a | 10.30±0.33b |  | 10.00±1.00b | 47.00±1.53a | 8.67±1.20b |  | 9.67±0.33b | 54.30±2.85a | 8.00±0.58b |

When the lowercase letters are the same for the elements’ concentrations within a sample type, this indicates that samples did not differ significantly among the oak species, Duncan’s multiple range test, *p* < 0.05.

Table S2 The simple stepwise multiple regressions of acorn infection rate against weevil size, and their full models.

| Methods | Equation | R^2^ | *p* |
| --- | --- | --- | --- |
| Full model | Infection rate = 21.89 + 4.48 × acorn weight + 176.29 × weevil weight + 3.16 × acorn N + 23.30 × acorn P | 0.85 | 0.05 |
| Stepwise | Infection rate = 3.97 + 69.74 × weevil weight | 0.70 | 0.005 |

**Table S3** Contrasting element concentrations of acorns and weevil larvae in *Q. variabilis* through subtropical sites and present study.

| Elements  (mg g^-1^) | Acorns | | | | | |  | Weevil larvae | | | | | |
| --- | --- | --- | --- | --- | --- | --- | --- | --- | --- | --- | --- | --- | --- |
|  | P-deficient sites | | P-rich sites | | This study | |  | P-deficient sites | | P-rich sites | | This study | |
|  | Mean | CV | Mean | CV | Mean | CV |  | Mean | CV | Mean | CV | Mean | CV |
| C | 440.98 | 5.21 | 438.75 | 5.90 | 441.23 | 0.08 |  | 633.08 | 7.23 | 639.57 | 5.30 | 620.37 | 1.64 |
| H | 59.92 | 7.62 | 63.14 | 5.77 | 63.97 | 0.99 |  | 101.94 | 7.86 | 104.64 | 5.42 | 94.80 | 2.00 |
| O |  |  |  |  | 455.76 | 0.51 |  |  |  |  |  | 219.70 | 3.13 |
| N | 7.53 | 23.12 | 7.05 | 15.76 | 6.40 | 8.13 |  | 53.64 | 9.20 | 55.88 | 9.21 | 50.20 | 12.31 |
| P | 1.00 | 15.86 | 1.21 | 16.21 | 1.22 | 8.37 |  | 9.38 | 7.56 | 10.03 | 5.32 | 3.95 | 5.73 |
| S | 0.69 | 29.40 | 0.66 | 24.07 | 0.66 | 25.30 |  | 3.53 | 21.65 | 3.51 | 21.65 | 2.17 | 0.92 |
| K | 7.54 | 9.14 | 7.88 | 8.89 | 8.20 | 8.82 |  | 12.41 | 6.52 | 12.50 | 8.44 | 6.02 | 7.58 |
| Na | 0.62 | 68.12 | 0.55 | 38.36 | 0.10 | 26.89 |  | 0.73 | 169.55 | 1.25 | 177.00 | 0.37 | 23.51 |
| Ca | 0.93 | 69.50 | 0.72 | 18.53 | 0.81 | 31.48 |  | 0.89 | 29.26 | 1.00 | 33.93 | 0.42 | 9.90 |
| Mg | 0.65 | 14.18 | 0.73 | 25.15 | 0.85 | 5.93 |  | 3.68 | 11.79 | 4.12 | 9.03 | 1.18 | 10.28 |
| Al | 0.12 | 177.38 | 0.06 | 68.44 | 0.01 | 23.55 |  | 0.10 | 106.30 | 0.20 | 101.34 | 0.03 | 62.39 |
| Fe | 0.06 | 92.30 | 0.07 | 49.98 | 0.02 | 26.32 |  | 0.13 | 75.62 | 0.34 | 118.54 | 0.05 | 43.27 |
| Mn | 0.12 | 53.85 | 0.05 | 63.44 | 0.07 | 44.57 |  | 0.10 | 25.62 | 0.08 | 15.15 | 0.04 | 19.05 |
| Zn | 0.06 | 263.44 | 0.02 | 68.78 | 0.01 | 5.97 |  | 0.13 | 30.33 | 0.12 | 25.89 | 0.05 | 9.08 |

The data of P-deficient and P rich sites was obtained from Ji et al. (2017) in subtropical China


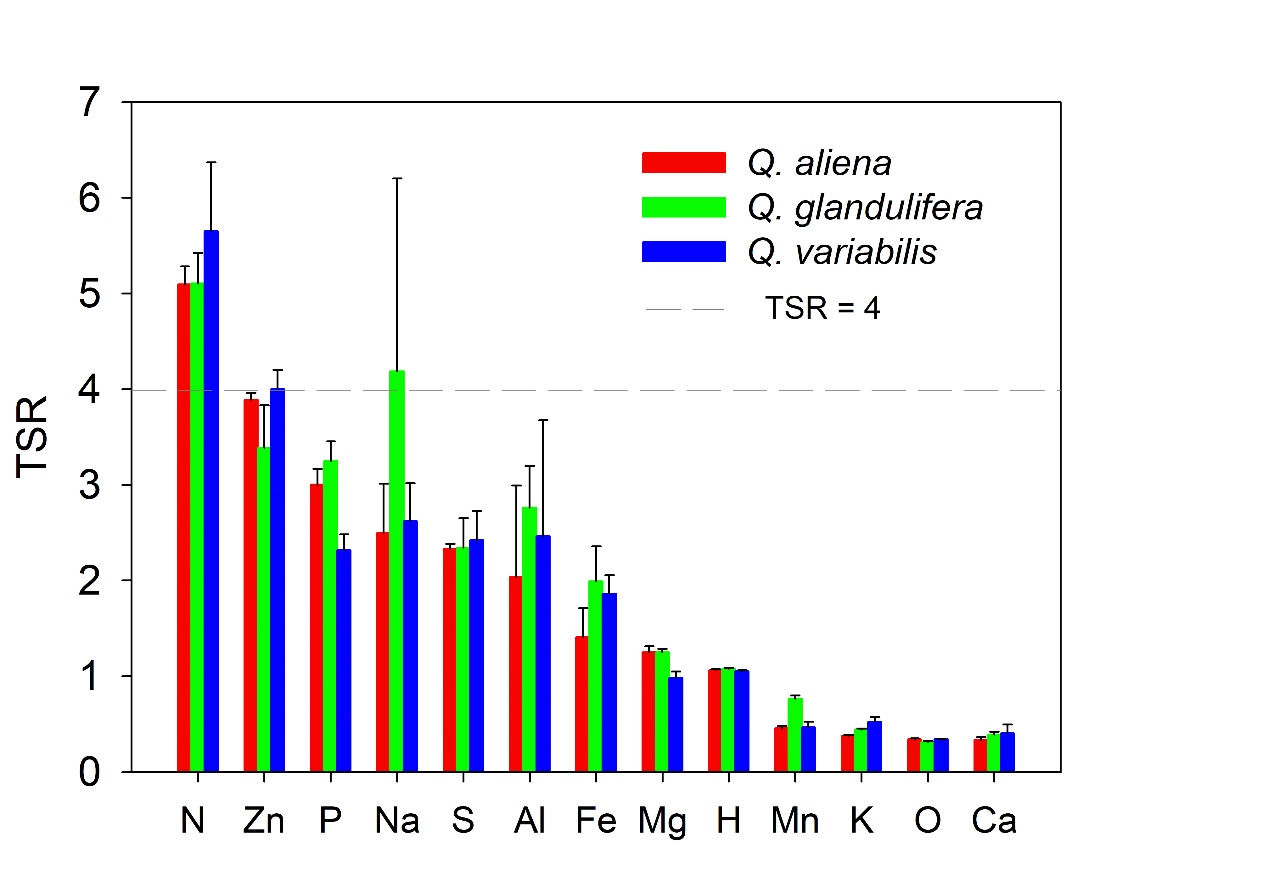


**Fig. S2.** The trophic stoichiometric ratio index based on three acorn species and weevil larvae The quantification of limiting elements may be done using the Trophic Stoichiometric Ratio Index (TSR) (Filipiak 2018). TSR = (C:X)_food_ / (C:X)_consumer,_ where C is the concentration of carbon and X is the concentration of element x. If the TSR ≥ 4.0 indicates the element x imposes a possible constraint on the growth and development of the consumer.

**References**

**Ji, H., B. Du, and C. Liu. 2017.** Elemental stoichiometry and compositions of weevil larvae and two acorn hosts under natural phosphorus variation. Sci. Rep. 7: 45810.

**Filipiak, M. 2018.** Chapter 13. Nutrient dynamics in decomposing dead wood in the context of wood eater requirements: The ecological stoichiometry of saproxylophagous insects, pp. 429-469. *In* M. D. Ulyshen (ed.), Saproxylic Insects*.* Springer International Publishing, Berlin, Germany.
